# Population Genetic Analysis of *Theileria annulata* from Six Geographical Regions in China, Determined on the Basis of Micro- and Mini-satellite Markers

**DOI:** 10.3389/fgene.2018.00050

**Published:** 2018-02-19

**Authors:** Fangyuan Yin, Zhijie Liu, Junlong Liu, Aihong Liu, Diaeldin A. Salih, Youquan Li, Guangyuan Liu, Jianxun Luo, Guiquan Guan, Hong Yin

**Affiliations:** ^1^State Key Laboratory of Veterinary Etiological Biology, Key Laboratory of Veterinary Parasitology of Gansu Province, Lanzhou Veterinary Research Institute, Chinese Academy of Agricultural Sciences, Lanzhou, China; ^2^Central Veterinary Research Laboratory, Khartoum, Sudan; ^3^Jiangsu Co-innovation Center for Prevention and Control of Important Animal Infectious Diseases and Zoonoses, Yangzhou, China

**Keywords:** *Theileria annulata*, micro- and mini-satellites, genetic diversity, population structure, China

## Abstract

*Theileria annulata*, a tick-borne apicomplexan protozoan, causes a lymphoproliferative disease of cattle with high prevalence in tropical and sub-tropical regions. Understanding the genetic diversity and structure of local populations will provide more fundamental knowledge for the population genetics and epidemics of protozoa. In this study, 78 samples of *T. annulata* collected from cattle/yaks representing 6 different geographic populations in China were genotyped using eight micro- and mini-satellite markers. High genetic variation within population, moderate genetic differentiation, and high level of diversity co-occurring with significant linkage disequilibrium were observed, which indicates there is gene flow between these populations in spite of the existence of reproductive and geographical barriers among populations. Furthermore, some degree of genetic differentiation was also found between samples from China and Oman. These findings provide a first glimpse of the genetic diversity of the *T. annulata* populations in China, and might contribute to the knowledge of distribution, dynamics, and epidemiology of *T. annulata* populations and optimize the management strategies for control.

## Introduction

Tropical theileriosis, one of the fatal tick-borne diseases, caused by *Theileria annulata* is transmitted by ticks of the genus *Hyalomma* and widespread in North Africa, southern Europe, India, the Middle East and Central Asia (Robinson, 1982). *T. annulata* was one of the most pathogenic protozoa infective to cattle, causing seriously economic losses attributed to the high morbidity and mortality (Gharbi et al., 2006). In addition, *T. annulata* infection can occur in a carrier state during theileriosis recovery, which leads to continual spreading of the diseases (Bishop et al., 2004). This parasite has a complex life cycle undergoing two phases, haploid asexual propagation in bovine hosts and diploid sexual generation in ticks (Gauer et al., 1995). Briefly, sporozoites are inoculated into the host by feeding ticks and rapidly invade leukocytes, then transformed into multinucleated schizonts that rupture and release un-nucleate schizoites. Lastly, the schizoites invade erythrocytes and develop into piroplasms. The ingested piroplasms from infected bovines by ticks survive in the gut where male and female gametes occur fusing to form zygotes and then differentiate into motile kinetes. The kinetes migrate to the salivary gland; ultimately, the bovine-infective sporozoites are produced (Dobbelaere and Heussler, 1999). In China, this disease formally was prevalent in 13 provinces, such as Xinjiang, Inner Mongolia, Ningxia, Gansu, Shaanxi, Shanxi, Heilongjiang, Jilin, Liaoning, Hebei, Shandong, Henan, and Hubei (Luo and Lu, 1997; Yin et al., 2008). Recently, along with the increase of demand for beef, many corporations strengthened the trade and transport of cattle across the great distance in this country (Xu et al., 2011). As a result, the infections of *T. annulata* were detected from non-epizootic areas, Guangdong, Chongqing, Hunan, Yunnan, and Guizhou (Yin et al., 2008; Liu et al., 2015; Abdallah et al., 2017).

The knowledge of genetic variation, population differentiation, and structures in protozoan parasites are crucial to understand their genotypic characteristics, evolution, and epidemiology (Majewska and Sulima, 1999). A number of genetic diversity studies of apicomplexan parasites have been investigated, including *Cryptosporidium parvum* (Mallon et al., 2003; Morrison et al., 2008), *Plasmodium falciparum* (Anderson et al., 2000; Ingasia et al., 2016), and *Toxoplasma gondii* (Su et al., 2012). These studies showed that the main factors determined population structures (clonal, panmictic, and epidemic) among different species could be related to their virulence, geographical distance, transmission intensity, and host preference. Neutral micro-satellite markers with high polymorphism, high mutation rate, and Mendelian mode of inheritance have been broadly applied to explore population genetics (Abdelkrim et al., 2009). The population diversity of *T. annulata* has been examined across different regions in the world, such as Oman, Portugal, Tunisia, and Turkey. A degree of genetic differentiation was detectable among these four populations. Conclusions from these studies indicated that high level of genetic variability was observed among populations and evidence for sub-structuring was found to connect with geographical distance in *T. annulata* (Weir et al., 2007, 2011; Al-Hamidhi et al., 2015; Gomes et al., 2016).

Although genetic structure of *T. annulata* was well known in Oman, Portugal, Tunisia, and Turkey (Weir et al., 2011; Al-Hamidhi et al., 2015; Gomes et al., 2016), the population genetic analysis in China is not yet studied. In this study, we explored the genetic diversity, population structure, and genetic differentiation of *T. annulata* from 6 geographical regions (4 epizootic areas and 2 non-epizootic areas) in China employing polymorphic micro- and mini-satellite markers. Moreover, a comparative analysis was performed with the Oman populations to investigate the genetic difference over a large geographical scale. Overall, the information will help us to know the distribution of the different *T. annulata* population and to adopt different measures to prevention and control this parasite.

## Materials and Methods

### Sample Collection and DNA Extraction

Animal experiment was approved by the Science and Technology Department of Gansu province, China (permit SYXK2010-0003). The 330 blood samples were randomly collected from cattle and yaks with no apparent clinical signs of theileriosis in Gansu (24 from cattle and 16 from yaks), Hainan (20 from cattle), Inner Mongolia (44 from cattle), Liaoning (38 from cattle), Tianjin (24 from cattle), and Xinjiang (164 from cattle) provinces of China (**Supplementary Figure S1**). The sample distributions cover from arid and semi-arid to sub-humid climate regions. Approximately 10 ml blood sample was collected from the jugular veins of each animal, and injected into EDTA vacutainers tubes. Genomic DNA was extracted from 300 μl thawed blood using the QIAamp DNA Mini Kit (Qiagen, Germany) according to the manufacturer’s instructions and then stored at -20°C until used.

### Screening the Positive Samples of *T. annulata*

To confirm the *T. annulata* positive samples, cytochrome b gene (*cytb*) was amplified using the specific primers AnCb-F (5′-CGGTTGGTTTGTTCGTCTTT-3′) and AnCb-R (5′-GCCAATGGA TTTGAACTTCC-3′) (Liu et al., 2015). PCR was performed in a final volume of 25 μl mixture containing 12.5 μl Premix Taq DNA polymerase (TaKaRa, China), 0.1 μM of primer pair and 2 μl of DNA template under the reaction conditions previously described by Liu et al. (2015). The DNA sample extracted from purified *T. annulata* merozoites was considered as positive control and the nuclease free water was used as negative control. Amplicons were analyzed and visualized under ultraviolet light, then all the positive products were sequenced directly using AnCb-F/AnCb-R primers and analyzed for further confirmation.

### Micro- and Mini-satellite Genotyping

Three micro-satellite markers (TS5, TS9, and TS12) and five mini-satellite markers (TS6, TS8, TS15, TS20, and TS25) were selected to detect the genotype of each sample based on the methods described by Weir et al. (2007). Each forward primer was labeled with fluorescein FAM at the 5′ ends. PCR was performed as above and optimized as described by Weir et al. (2007). Amplified products were separated by capillary electrophoresis in an ABI 3730 XL analyzer (Applied Biosystems, United States) with LIZ500 as the internal size standard and alleles were scored using the GeneMarker software (SoftGenetics, United States). Only minor peaks which are larger than 33% of the maximum peak at height were recorded. For obtaining the most abundant genotypes of each sample, the predominant allele was identified per locus and the data was combined to generate a multilocus genotype (MLG) (Weir et al., 2007). Then, the obtained MLGs were compared with the dataset from previous study in Oman (Al-Hamidhi et al., 2015).

### Data Analysis

Genetic diversity parameters such as the number of different alleles (*Na*), number of effective alleles (*Ne*), and unbiased heterozygosity (*uh*) were calculated by the program GenAlex 6.5 (Peakall and Smouse, 2012). The heterozygosity of *T. annulata* cannot be directly observed as the blood stage of *T. annulata* is haplotypic. Thus, expected heterozygosity (*He*) was calculated to measure genetic variation for each population. Rarefied allelic richness and private alleles were calculated to correct for sample size using the program HP-rare (Kalinowski, 2005). The multiplicity of infection (MOI), which could be determined by the number of different alleles detected within each sample, was calculated for each sample across the eight loci and the average of MOI for each population was then calculated. Pairwise *F*_ST_ values were conducted with Arlequin 3.5 to estimate the genetic differentiation between pairs of populations (Excoffier and Lischer, 2010). Analysis of molecular variance (AMOVA) was performed using the same program (Arlequin 3.5) to assess the population variation in breed differences. Mantel test was conducted to determine the relationship of geographical and genetic distance using Arlequin 3.5. For comparing the genetic relationships between China and Oman, principal coordinate analysis (PCoA) was applied by GenAlex 6.5 (Peakall and Smouse, 2012). Population structure analysis was investigated by STRUCTURE v2.3.3 (Pritchard et al., 2000) using the admixture ancestry model. The number of clusters (*K*) was explored from 1 to 10 with 6 times replications per *K* employing Bayesian Markov Chain Monte Carlo (MCMC) approach. To further confirm the genetic clusters, Discriminant Analysis of Principal Components (DAPC) was performed by the *adegenet* package (Jombart, 2008) implemented in the R software (R Development Core Team, 2009). DAPC, a without *a priori* method, is to test population differentiation. For DAPC, the best number of groups was assessed using the function “*find.clusers*,” which runs *K*-means with increasing *K*-values. Bayesian Information Criterion (BIC) was applied to compare the different groups, and the lowest BIC value used to infer the ideal number of groups (Jombart et al., 2010).

The null hypothesis of linkage equilibrium (LE) in each *T. annulata* isolate from China, and between China and Oman, is assessed using the standard index of association (*I*_A_^S^) which is conducted by the program LIAN version 3.7 (Haubold and Hudson, 2000). Variance *V*_D_ is computed from the distribution of mismatch values, and then is compared with the variance expected (*V*e) for LE. Monte Carlo simulation or a parametric test is employed to test the null hypothesis that *V*_D_ = *V*e, and the results provide 95% confidence limits, denominated as *L*_MC_ and *L*_Para_, respectively. When *V*_D_ is observed greater than *L*, the null hypothesis is discarded and linkage disequilibrium (LD) is accepted.

## Results

### PCR Screening

In total, 124 (39.49%, 95% CI: 0.34–0.45) cattle (Gansu 14, Hainan 11, Inner Mongolia 12, Liaoning 21, Tianjin 9, and Xinjiang 57) and 6 (37.5%, 95% CI: 0.14–0.61) yak blood samples were positive employing AnCb-F/AnCb-R primers. Out of the 130 samples, 78 samples were successfully genotyped using the eight micro- and mini-satellite markers. A total of 52 samples were excluded due to the failed amplification by most of the markers. From the 78 *cytb* amplicons, 5 distinct haplotypes were obtained after sequence editing and alignment (GenBank accession numbers MG735205–MG735209).

### Genetic Diversity and Multiplicity of Infection

All the markers were highly polymorphic, polymorphic information content (PIC) varying from 0.625 to 0.902, and the number of alleles ranging from 8 (TS20) to 21 (TS12) at each locus (**Supplementary Table S1**). The number of alleles per population ranged from 2.38 (± 0.32) in Tianjin to 8.50 (± 1.00) in Xinjiang, and the unbiased expected heterozygosity (*He*) per population from 0.46 (± 0.13) in Liaoning to 0.82 (± 0.04) in Xinjiang with mean *He* 0.64 (± 0.05) indicating high diversity across all the six populations in China (**Table 1**). For each population, allelic richness (*Ar*) ranged from 2.68 to 5.00, and the private alleles (*Ap*) ranged from 0.44 to 2.58 (**Table 1**). The population genetic characteristics analysis revealed that Xinjiang population showed more diversity than other populations of *T. annulata*.

**Table 1:** Population genetic data at eight micro- and mini-satellite loci for six *Theileria annulata* isolates in China.

Population	*N*	*Na*	*Ne*	*Ar*	*Ap*	*He*	*uh*
Xinjiang	23	8.50	5.43	5.00	2.58	0.78	0.82
Liaoning	19	3.00	2.16	2.68	0.44	0.39	0.46
Gansu	11	2.50	2.12	3.03	0.71	0.39	0.50
Inner Mongolia	8	3.13	2.51	4.00	0.93	0.51	0.64
Tianjin	8	2.38	2.13	3.38	0.75	0.46	0.71
Hainan	9	2.75	2.45	3.42	1.16	0.49	0.70
All	78	3.71	2.80	3.59	1.10	0.50	0.64


**N*: number of individuals; *Na*: number of alleles; *Ne*: number of effective alleles; *Ar*: Allelic richness; *Ap*: private alleles; *He*: expected heterozygosity; *uh*: unbiased heterozygosity.*

Out of the 78 samples, 55 expressed multiple genotypes, representing a mix infection. MLG data of Gansu population were all from yaks, and Hainan, Inner Mongolia, Liaoning, Tianjin, and Xinjiang five populations were from cattle. The mean numbers of alleles across the eight loci for each isolate were calculated to estimate the MOI within each isolate (**Table 2**). All the populations in China reported average 1.61 alleles per locus and ranged from 1.33 to 1.85. The results from MOI showed that Xinjiang population was observed with more variation with a maximum of 2.75, whereas the population from Tianjin possessed a minimum value of 1.50 indicating less variation.

**Table 2:** Multiplicity of infection in six *Theileria annulata* isolates in China.

Population	*N*	Number of allele per locus per isolate			
		
		Mean	Minimum	Maximum	*SD*
Xinjiang	21	1.85	1.14	2.75	0.38
Liaoning	11	1.39	1.25	1.67	0.16
Gansu	6	1.80	1.40	2.20	0.23
Inner Mongolia	6	1.53	1.13	2.67	0.52
Tianjin	6	1.33	1.17	1.50	0.11
Hainan	5	1.35	1.25	1.67	0.16
All	55	1.61	1.13	2.75	0.39


**N*: number of individuals, SD: standard deviation.*

### Linkage Disequilibrium Analysis

Extent of LD was measured to estimate whether the *T. annulata* populations across different areas occurred in panmixia with high level of gene flow using the standard index of association (*I*_A_^S^). No evidence for random mating was found when the six *T. annulata* populations from China were considered as one population. *I*_A_^S^ value of 0.1224 was positive, and *V*_D_ (2.6253) was tested greater than *L*_MC_ (1.5727) and *L*_Para_ (1.5635) indicating LD (*p* < 0.0001) (**Table 3**). When each region was treated separately in China, LE was observed in Gansu, Inner Mongolia, Tianjin, Hainan, and Xinjiang five populations, while LD was found in Liaoning (**Table 3**). When combining China and Oman populations, although the *I*_A_^S^ value (0.0569) was lower, LD was detected (**Table 3**).

**Table 3:** Linkage equilibrium analyses among *Theileria annulata* populations.

Country	Region	*N*	*I*_A_^S^	*V*_D_	*L*_MC_	*L*_Para_	Linkage
China		55	0.1224	2.6253	1.5727	1.5635	LD
	Xinjiang	21	0.0161	1.2303	1.3164	1.2992	LE
	Liaoning	11	0.1268	3.1414	2.6970	2.5181	LD
	Gansu	6	0.0027	0.9238	1.6381	1.4046	LE
	Inner Mongolia	6	0.0123	1.4286	2.2857	2.0887	LE
	Tianjin	6	0.0422	2.0952	3.2381	2.7827	LE
	Hainan	5	-0.0308	1.0667	2.8444	2.3459	LE
China and Oman		286	0.0569	1.1364	0.8321	0.8318	LD


**N*: number of individuals, *I*_*A*_^*S*^: standard index of association, *V*_*D*_: mismatch variance (linkage analysis), *L*_*MC*_: upper 95% confidence limits of Monte Carlo simulation, *L*_*Para*_: parametric tests, LD: linkage disequilibrium, LE: linkage equilibrium.*

### Population Genetic Differentiation

The levels of genetic differentiation among the six populations were shown in **Table 4**. Most *F*_ST_ values demonstrated that moderate genetic differentiation was found between pairs of populations ranging from -0.020 to 0.264. However, high genetic differentiation was observed between Gansu and other populations with *F*_ST_ values ranging from 0.158 to 0.264 (> 0.150). Meanwhile, relatively high genetic differentiation was obtained between Xinjiang and Liaoning, and low differentiation was between Tianjin and Liaoning populations (**Table 4**).

**Table 4:** Pairwise *F_*ST*_* between *Theileria annulata* populations from six geographical regions of China.

Population	Xinjiang	Liaoning	Gansu	Inner Mongolia	Tianjin	Hainan
Xinjiang						
Liaoning	0.249^∗∗∗^					
Gansu	0.239^∗∗∗^	0.264^∗∗∗^				
Inner Mongolia	0.093^∗∗^	0.102^∗∗∗^	0.158^∗∗∗^			
Tianjin	0.186^∗∗∗^	-0.020	0.196^∗∗∗^	-0.005		
Hainan	0.129^∗∗∗^	0.145^∗∗∗^	0.229^∗∗^	0.065^∗^	0.066	


*^∗^*p* < 0.05; ^∗∗^*p* < 0.01; ^∗∗∗^*p* < 0.001.*

Between the breed groups, AMOVA showed that most of the genetic variation (79.01%) was mainly partitioned within populations rather than among groups and among populations within groups, as determined by low values of *F*_CT_ (0.057) and *F*_SC_ (0.162) (**Table 5**).

**Table 5:** Analysis of molecular variance (AMOVA) of *Theileria annulata* populations.

Variance component	Variance	% of total	*F*-statistics	*p*-value
Among groups^a^	0.191	5.74	*F*_CT_ = 0.057	0.344
Among populations within groups	0.509	15.25	*F*_SC_ = 0.162	<0.0001^∗^
Within populations	2.634	79.01	*F*_ST_ = 0.210	<0.0001^∗^


*^*a*^Population variance was divided into two groups according to the breed differences of host, including cattle group (Hainan, Inner Mongolia, Liaoning, Tianjin, and Xinjiang) and a yak group (Gansu). ^∗^*p* < 0.0001.*

### Population Structure

**FIGURE 1:**
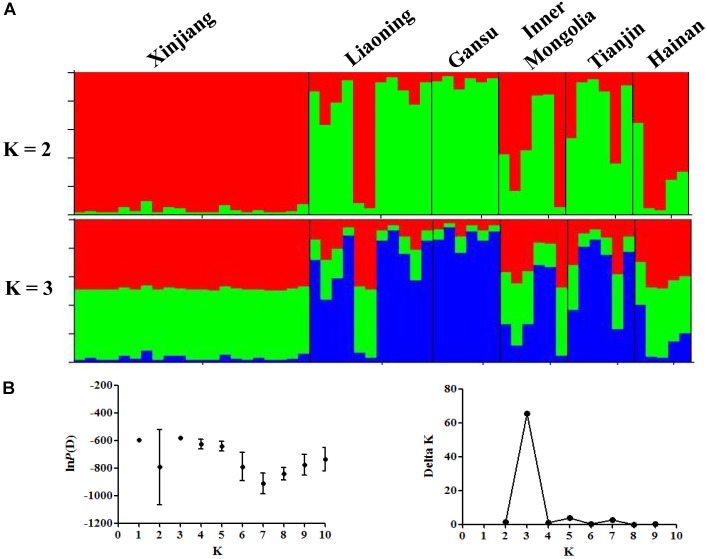
Bayesian clustering analysis among *Theileria annulata* populations. **(A)** The clusters are *K* = 2 and *K* = 3, colors represent proportions in each of the *K* inferred clusters and sampling locations are denoted. **(B)** Rate of change of the ln*P*(D) (mean ± SD) for *K*-values from 1 to 10. The true number of clusters is 3 according to the calculation of delta *K*.

**FIGURE 2:**
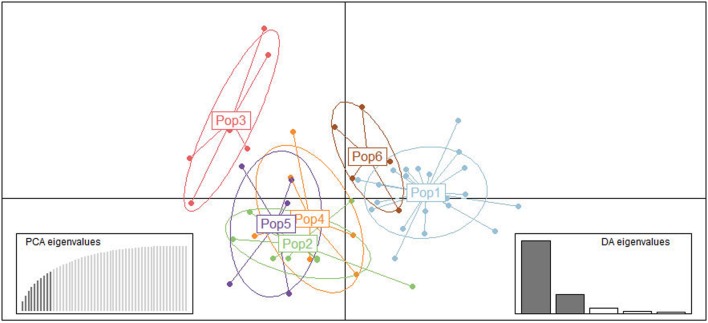
Genetic structure of *Theileria annulata* populations in China based on discriminant analysis of principal components (DAPC). Each population is pointed out by different color symbols. Pop1: Xinjiang; Pop2: Liaoning; Pop3: Gansu; Pop4: Inner Mongolia; Pop5: Tianjin; Pop6: Hainan.

The results of STRUCTURE analysis showed that the members from six populations were assigned to different clusters indicating weak population structure among populations (**Figure 1A**). Bayesian clustering analysis showed that optimal number of clusters was observed at *K* = 3 (**Figure 1B**), revealing that all the *T. annulata* populations could be divided into three subgroups (Cluster 1, Cluster 2, and Cluster 3). Obvious mixture haplotypes were found in all populations. Deep analysis revealed that the members from Gansu population were assigned to the Cluster 3 with the highest proportion (87.7%), and the members from Xinjiang population were assigned to the Cluster 1 and Cluster 2 with same rate (48.5%). DAPC analysis supported the division of *T. annulata* population into three groups (**Figure 2**). These findings indicate that a low genetic sub-structuring exists in *T. annulata* populations.

For the six *T. annulata* populations, the results of the relationship between genetic and geographical distance demonstrated that the genetic differentiation of these populations do not respond to isolation by distance pattern (Mantel test: *p* = 0.164).

**FIGURE 3:**
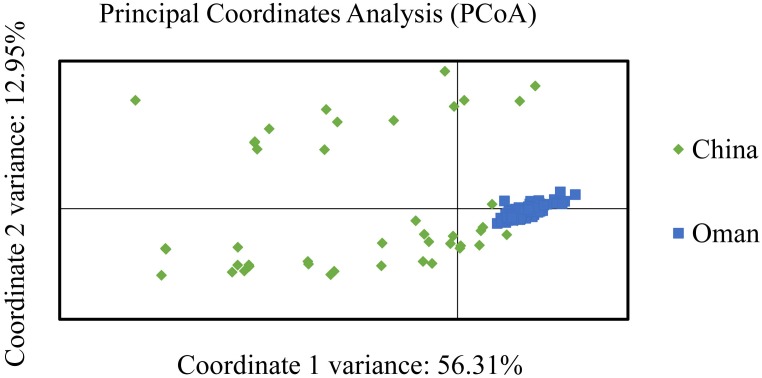
Principal coordinate analysis (PCoA) of *Theileria annulata* populations from China and Oman.

The PCoA analysis supported a high degree of genetic differentiation between populations in China and Oman (**Figure 3**). Most of the samples from China and Oman were classified into different quadrants revealed that an obvious geographic sub-structuring was detected in association with independent of geographic origin.

## Discussion

In the present study, the genetic diversity of *T. annulata* in China was slightly lower in comparison with other endemic countries (Turkey, Tunisia, and Oman), but a similar level with Portugal (Weir et al., 2011; Al-Hamidhi et al., 2015; Gomes et al., 2016). Sample size is a crucial parameter affecting the genetic diversity in a population; the obvious differences of *He* index between these studies could be explained by the small sample size used in this study (McDonald and Linde, 2002).

The high prevalence of MOI in Chinese *T. annulata* populations was consistent with other apicomplexan parasites such as *C. parvum*, *P. falciparum*, *T. parva*, and *T. lestoquardi* (Muleya et al., 2012; Cacciò et al., 2015; Wei et al., 2015; Al-Hamidhi et al., 2016). Such multiple infections could be related to transmission intensity, and the rate of new infections and the duration of infections as reported in *P. falciparum* (Pinkevych et al., 2015). Comparison between populations revealed, the frequency of mixed genotypes was relatively high in Xinjiang, Gansu, and Inner Mongolia supported by the fact that these are the epidemic areas of tropical theileriosis in China (Luo and Lu, 1997). Therefore, it could be inferred that the differences in multiple infections might be relevant to transmission intensity and the amount of tick-borne vectors in different sampling sites. Although the determinants of the MOI are unclear, further works are required to explore MOI, which is a crucial parameter for cross fertilization and recombination among the same or different genotypes (Al-Hamidhi et al., 2015).

When China and Oman populations were combined, significant LD was found indicating the existence of low level of genetic exchange, population differentiation, and geographical sub-structuring between the populations from these two countries. The genetic differentiation and sub-structuring were supported by PCoA analysis (**Figure 3**), which showed the samples from China and Oman formed completely discrete clusters. Geographical barriers along with the trade barriers and local agricultural policies greatly restricted host movement, which might be a reasonable explanation for this genetic subdivision, the reduction of gene flow and the formation of isolated populations between different countries (Weir et al., 2007). Many similar results were found between populations in Oman, Portugal, Tunisia, and Turkey, and these indicated that geographical differentiation exist in different countries (Weir et al., 2007, 2011; Al-Hamidhi et al., 2015; Gomes et al., 2016). Although significant LD was also found across the entire Chinese population, LE was observed in five of the six *T. annulata* populations when each region was calculated independently. These findings suggested that variation in effective population size and population subdivision could be corroborated with the above differences (Waples and England, 2011). The detection of LE in four populations from Gansu, Inner Mongolia, Tianjin, and Hainan is questionable, since the sample size is small. LE in the Xinjiang population indicated that association of alleles and random mating occurred. DAPC analysis was further proved the existence of population substructure in Xinjiang (**Figure 2**). Moreover, despite the high MOI and abundant diversity, significant LD was observed in the overall Chinese population. Inbreeding might be a more realistic explanation for the inconsistent results. Such similar observations of strong LD and high diversity are in agreement with *T. parva*, *P. falciparum*, and *P. vivax* (Muleya et al., 2012; Wei et al., 2015; Hong et al., 2016).

Moderate genetic differentiation was detected between the six populations in China (*F*_ST_ > 0.05). High genetic differentiation was observed between the Gansu population and others (*F*_ST_ values between 0.158 and 0.264), which could be related to the reproductive isolation. The obvious differences between samples from different host were confirmed by the high pairwise *F*_ST_ values and DAPC analysis. Furthermore, the yak is a special breed which was collected from the Tibetan Autonomous County in Gansu. The limited host movement and reproductive isolation might obscure the genetic exchange (Ramiro et al., 2015). The population from Xinjiang also showed a high genetic differentiation (*F*_ST_ values between 0.093 and 0.249) in comparison with other populations. The low level of gene flow with other populations might be influenced by the nomadic lifestyle, local agricultural policy, and the limited trade with other regions of China. Xinjiang, the largest land area in China, is far away from other sampling sites (about from 2721 to 5073 km). A fact is that Xinjiang belongs to an arid and semi-arid region with temperate continental climate, where is suitable for *Hyalomma* species development. Therefore, the specific geographical environment and ecological characteristic might restrict the opportunities to media transmission, genetic recombination, and genetic exchange of *T. annulata* with other populations in China. In our case, the special geographical location, the life way of people and animals, and the climatic features would explain why the Xinjiang population appears to be high genetic differentiation.

Despite the existence of reproductive and geographical barriers, the existence of the mixture haplotypes and the majority (79.01%) of the genetic variations within populations revealed that there were gene flow between these populations due to the movement of animals (Zhang et al., 2009). This finding could be supported by Bayesian and DAPC analysis. As expected, the Mantel test indicated that there was no correlation between genetic differentiation and geographic distances. The frequent movement of cattle may cause the migration of diverse parasites into different regions, and that results in a complex population structure. It is comprehensively considered that many important factors such as transmission intensity, host factors, and the discrepancy of geographical conditions caused particular genetic divergence and epidemic patterns in Chinese *T. annulata* populations.

## Conclusion

We compared the genetic variations between sympatric *T. annulata* in China for the first time. These results implied that moderate genetic differentiation, high degree of diversity, and significant LD were exhibited, and regional level of geographical sub-structuring was present in Chinese *T. annulata* populations. Furthermore, it indicated low levels of genetic exchange and obvious population subdivision between China and Oman at a large distance. This study not only improves the understanding of population structure of *T. annulata*, but also contributes to better management strategies for bovine theileriosis in China.

## Author Contributions

HY and GG conceived the project. ZL, JuL, AL, YL, GL, and JiL collected the samples. FY performed the experiment and data analysis. FY and DS interpreted the data. FY prepared the manuscript with the support from DS and GG. All authors read and approved the final version of the manuscript.

## Conflict of Interest Statement

The authors declare that the research was conducted in the absence of any commercial or financial relationships that could be construed as a potential conflict of interest.

## Supplementary Material

**FIGURE S1 |** Locations of six *Theileria annulata* populations in China. Samples from Hainan (*n* = 20), Inner Mongolia (*n* = 44), Liaoning (*n* = 38), Tianjin (*n* = 24), and Xinjiang (*n* = 164) were collected from cattle. Samples from Gansu were collected from cattle (*n* = 24) and yaks (*n* = 16).

**TABLE S1 |** Characteristics of the 8 micro- and mini-satellite loci used in six *Theileria annulata* populations from China.
