# Supplementary figures and images for: Population Genetic Analysis of Theileria annulata from Six Geographical Regions in China, Determined on the Basis of Micro- and Mini-satellite Markers
Source: Front Genet. 2018 Feb 19;9:50. doi: 10.3389/fgene.2018.00050 (PMC5826064; doi:10.3389/fgene.2018.00050)

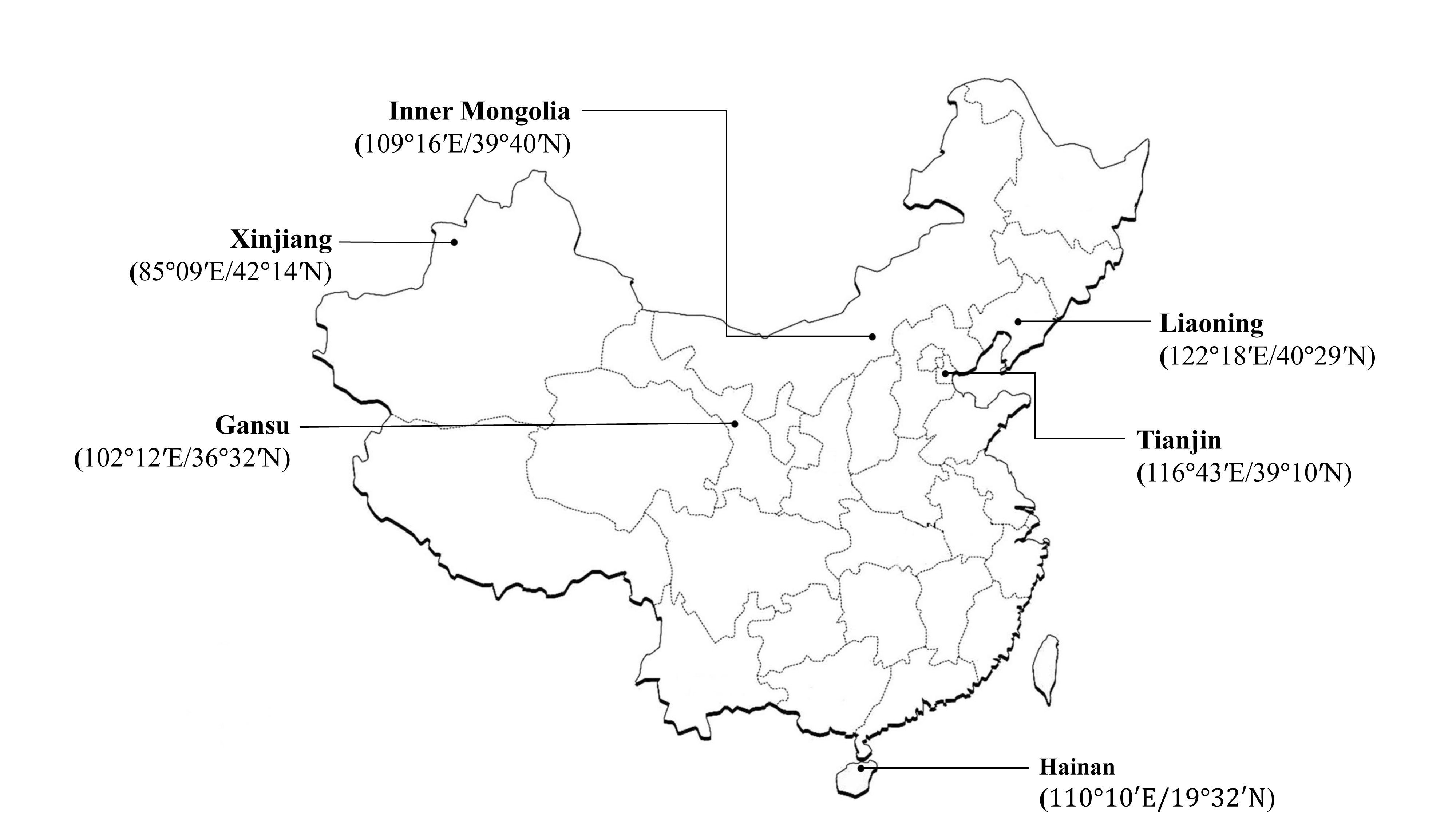

Supplement: Supplementary file 1 [file Image_1.JPEG]
