# Supplementary material for: Population Genetic Analysis of Theileria annulata from Six Geographical Regions in China, Determined on the Basis of Micro- and Mini-satellite Markers
Source: Front Genet. 2018 Feb 19;9:50. doi: 10.3389/fgene.2018.00050 (PMC5826064; doi:10.3389/fgene.2018.00050)
Supplement: Supplementary file 2 [file Table_1.DOCX]

**TABLE S1.** Characteristics of the 8 micro- and mini-satellite loci used in six *Theileria annulata* populations from China.

| Locus | *Na* | *He* | *PIC* |
| --- | --- | --- | --- |
| TS5 | 10 | 0.688 | 0.625 |
| TS6 | 17 | 0.929 | 0.890 |
| TS8 | 16 | 0.713 | 0.686 |
| TS9 | 12 | 0.732 | 0.682 |
| TS12 | 21 | 0.941 | 0.902 |
| TS15 | 17 | 0.849 | 0.808 |
| TS20 | 8 | 0.749 | 0.686 |
| TS25 | 9 | 0.828 | 0.772 |

*Na*: number of alleles, *He*: expected heterozygosity, *PIC*: polymorphism information content.
